# A Digital Home-Based Health Care Center for Remote Monitoring of Side Effects During Breast Cancer Therapy: Prospective, Single-Arm, Monocentric Feasibility Study

**DOI:** 10.2196/64083

**Published:** 2025-05-02

**Authors:** Hanna Huebner, Lena A Wurmthaler, Chloë Goossens, Mathias Ernst, Alexander Mocker, Annika Krückel, Maximilian Kallert, Jürgen Geck, Milena Limpert, Katharina Seitz, Matthias Ruebner, Philipp Kreis, Felix Heindl, Manuel Hörner, Bernhard Volz, Eduard Roth, Carolin C Hack, Matthias W Beckmann, Sabrina Uhrig, Peter A Fasching

**Affiliations:** 1 Department of Gynecology and Obstetrics, Universitätsklinikum Erlangen, Friedrich-Alexander-Universität Erlangen-Nürnberg (FAU), Universitätsstraße 21/23, Erlangen, 91054, Germany, 49 9131 8533553; 2 Bavarian Cancer Research Center, Erlangen, Germany; 3 Comprehensive Cancer Center Erlangen-EMN, Erlangen, Germany; 4 REFINIO GmbH, Rohr, Germany; 5 Nuremberg Institute of Technology Georg Simon Ohm, Nuremberg, Germany

**Keywords:** breast cancer, digital medicine, telehealth, remote monitoring, cyclin-dependent kinase 4/6 inhibitor, CDK4/6 inhibitor, mobile phone

## Abstract

**Background:**

The introduction of oral anticancer therapies has, at least partially, shifted treatment from clinician-supervised hospital care to patient-managed home regimens. However, patients with breast cancer receiving oral cyclin-dependent kinase 4/6 inhibitor therapy still require regular hospital visits to monitor side effects. Telemonitoring has the potential to reduce hospital visits while maintaining quality care.

**Objective:**

This study aims to develop a digital home-based health care center (DHHC) for acquiring electrocardiograms (ECGs), white blood cell (WBC) counts, side effect photo documentation, and patient-reported quality of life (QoL) data.

**Methods:**

The DHHC was set up using an Apple Watch Series 6 (ECG measurements), a HemoCue WBC DIFF Analyzer (WBC counts), an iPhone SE (QoL assessments and photo documentation), a TP-Link M7350-4G Wi-Fi router, and a Raspberry Pi 4 Model B. A custom-built app stored and synchronized remotely collected data with the clinic. The feasibility and acceptance of the DHHC among patients with breast cancer undergoing cyclin-dependent kinase 4/6 inhibitor therapy were evaluated in a prospective, single-arm, monocentric study. Patients (n=76) monitored side effects—ECGs, WBC counts, photo documentation, and QoL—at 3 predefined time points: study inclusion (on-site), day 14 (remote), and day 28 (remote). After the study completion, patients completed a comprehensive questionnaire on user perception and feasibility. Adherence to scheduled visits, the success rate of the data transfer, user perception and feasibility, and the clinical relevance of remote measurements were evaluated.

**Results:**

Mean adherence to the planned remote visits was 63% on day 14 and 37% on day 28. ECG measurements were performed most frequently (day 14: 57/76, 75%; day 28: 31/76, 41%). The primary patient-reported reason for nonadherence was device malfunction. The expected versus the received data transfer per patient was as follows: ECGs: 3 versus 3.04 (SD 1.9); WBC counts: 3 versus 2.14 (SD 1.14); QoL questionnaires: 3 versus 2.5 (SD 1.14); and photo documentation: 6 versus 4.4 (SD 3.36). Among patients, 81% (55/68) found ECG measurements easy, 82% (55/67) found photo documentation easy, and 48% (33/69) found WBC measurements easy. Additionally, 61% (40/66) of patients felt comfortable with self-monitoring and 79% (54/68) were willing to integrate remote monitoring into their future cancer care. Therapy-induced decreased neutrophil count was successfully detected (*P*<.001; mean baseline: 4.3, SD 2.2, ×10^9^/L; on-treatment: 1.8, SD 0.8, ×10^9^/L). All-grade neutropenia and corrected QT interval prolongations were detected in 80% (55/68) and 2% (1/42) of patients, respectively.

**Conclusions:**

Adherence to scheduled remote visits was moderate, with nonadherence primarily attributed to device-related complications, which may have also affected the success rate of data transfer. Overall, patients considered remote monitoring useful and feasible. The prevalence of reported adverse events was comparable to existing literature, suggesting clinical potential. This initial feasibility study highlights the potential of the DHHC.

## Introduction

Systemic therapies, such as chemotherapy, targeted therapies, or immunotherapy, are accompanied by several side effects that require continuous and regular monitoring. Monitoring of side effects is particularly important for treatment approaches involving oral medications, as these medications are usually administered at home. Side effect monitoring enables the early detection and prevention of adverse events and is crucial for treatment benefits and adherence to the treatment schedule [1].

In recent years, highly effective oral therapeutic options, such as cyclin-dependent kinase 4 and 6 inhibitors (CDK4/6i), have been introduced for the treatment of patients with hormone receptor-positive breast cancer. Even though these CDK4/6is are administered orally, the associated side effects, such as neutropenia, leukopenia, and corrected QT interval (QTc) prolongation, necessitate regular hospital appointments [2-10]. For patients in rural areas, such appointments present unique challenges. Due to a lack of nearby medical facilities, patients often have to travel long distances to receive adequate medical care, which can be physically and emotionally taxing. If these patients do not receive comprehensive cancer care, including side effect monitoring, they may experience delayed detection and treatment of serious adverse events, potentially affecting their quality of life (QoL), and survival outcomes [11-15]. Remote monitoring and eHealth options may be particularly valuable in addressing these challenges [16].

Remote, home-based monitoring using eHealth options such as apps, wearables, or mobile medical devices can allow health care providers to monitor patients’ health status and potential side effects in real time. Multiple studies have shown that remote monitoring of cancer treatment symptoms is linked to improved QoL, fewer treatment disruptions, and increased survival rates [13,17-19]. However, most of these studies included only remote patient-reported outcome assessments. Recently, home- and sensor-based technologies, including various wearable devices, have also been shown to be suitable tools for cancer care. For example, smartwatches and fitness trackers have been used to promote physical activity and monitor heart rate [20-23].

Even though remote monitoring systems, eHealth apps, and wearable devices have the potential to improve cancer care, several challenges still need to be addressed. In particular, while current smartwatch technologies can monitor heart rate and record Food and Drug Administration–approved electrocardiograms (ECGs), and several apps on the market can be used to document patient-reported outcomes, assess QoL, and track side effects, using these individual tools alone is not sufficient to provide comprehensive medical care at home. Therefore, we aimed to establish a digital home-based health care center (DHHC) that includes a smartphone to assess QoL and document visual side effects, a smartwatch to record ECGs, and a white blood cell (WBC) system to analyze a patient’s WBCs from capillary blood. The primary focus of this study was to assess the feasibility and acceptance of such a digital remote system for cancer care in order to tailor a patient-centered solution and improve access to quality care.

## Methods

### Study Design

The SMILER study (“Smart and Interactive Home-Based Health Care Project—A Digital Healthcare Feasibility Pilot Study Including the d.H2C2 Initiative”) was a monocentric, single-arm study with the primary objective of assessing the feasibility of remote WBC and ECG measurements, as well as data transmission of remote measurements using a DHHC. The study was conducted at the Department of Gynecology and Obstetrics at the University Hospital Erlangen (Universitätsklinikum Erlangen) in Germany.

Inclusion criteria were an indication for or current treatment with a CDK4/6i (regardless of cycle number) and an age of 22 years or older (in accordance with the minimum age requirements for the use of DHHC devices as specified by their respective manufacturers). Patients could not be included if they had pacemakers or implantable cardioverter defibrillators, severe blood coagulation disorders, abnormalities in the last known ECG, or other comorbidities that might impact at-home measurements. The study was conducted between October 2021 and December 2022.

CDK4/6i therapy was administered according to the Summary of Product Characteristics. All patients who had an indication for CDK4/6i therapy, as determined by the treating physician, or who were already receiving CDK4/6i therapy, were screened for the SMILER study. In general, palbociclib was started at 125 mg/day, and if necessary, reduced to 100 or 75 mg/day. Ribociclib was started at 600 mg/day, and if required, reduced to 400 mg/day and subsequently to 200 mg/day. Abemaciclib was initiated at 300 mg/day, with potential dose reductions to 200 and 100 mg/day.

**Figure 1.**
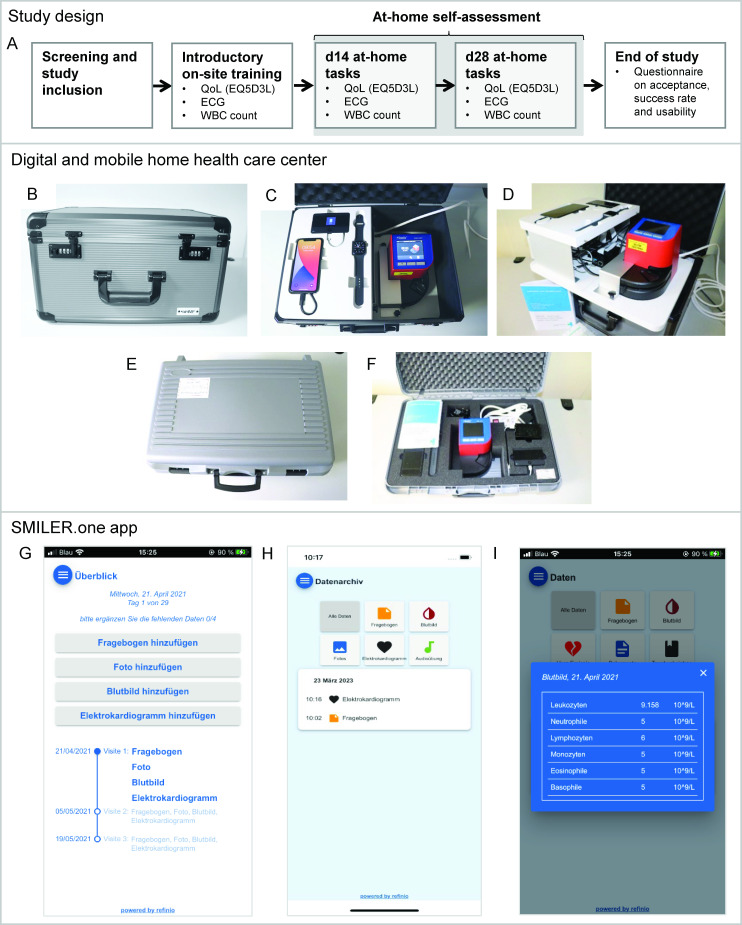
SMILER study (“Smart and Interactive Home-Based Health Care Project—A Digital Healthcare Feasibility Pilot Study Including the d.H2C2 Initiative”) design and technology setup. (A) The SMILER study included an initial training session, followed by 2 scheduled at-home tasks on day 14 (d14) and day 28 (d28). (B) Patients received a large case with (C) integrated charging for all devices, (D) specifically designed for at-home use, (E,F) or a smaller and lighter case with foam material to securely hold all devices. (G) The associated SMILER.one app featured a home screen displaying the trial tasks, (H) a data archive for storing all collected data, and (I) functionality for collecting and visualizing specific parameters such as WBC counts. ECG: electrocardiogram; QoL: quality of life; WBC: white blood cell.

After study inclusion, participants received the DHHC along with an initial introductory training. Study-relevant measurements were scheduled at study inclusion (on-site), day 14 (d14—remote), and day 28 (d28—remote; Figure 1A). At each of these time points, WBC counts, ECGs, and QoL (Q-5D-3L questionnaire) were monitored. Additionally, photo documentation of the ankle (as an exploratory subproject for the capturing of 3D photo data) was included. The ankle was chosen as an accessible location for photo acquisition where peripheral edema, a known side effect of CDK4/6i therapy, could be detected.

All participants continued their routine treatment and attended scheduled clinical visits. After the study had been completed, participants filled out a paper questionnaire on the acceptance, success rate, and usability of the DHHC. The SMILER study concluded after the predefined number of patients had been enrolled.

### Ethical Considerations

The study was conducted in accordance with local guidelines and regulations. Ethical approval was obtained from the Ethics Committee of the Friedrich-Alexander Universität Erlangen-Nürnberg (April 1, 2020: 47_20B). The original protocol was amended on March 22, 2022, to also include patients already receiving CDK4/6i therapy, as previously, patients could only be enrolled in the SMILER study at the start of CDK4/6i therapy. This change was implemented to improve study enrollment. All participants provided written informed consent before participation. Participants did not receive any form of compensation. Data were collected in a pseudonymized manner.

### Outcomes

The outcomes of the study were: adherence (primary objective), success rate of the data transfer, usability and feasibility of the DHHC, and clinical relevance. Adherence to scheduled study visits was assessed as the percentage of patients who completed the prescheduled measurements (±2 d around the scheduled visit). The success rate was evaluated as the number of remotely transferred measurements relative to the number of expected measurements. Feasibility was assessed based on the number of enrolled versus screened patients. Furthermore, patient-reported perception and usability were evaluated using a comprehensive paper-based questionnaire at study completion, which included Likert-scale questions on perceived usability. Usability was further assessed using the System Usability Scale (SUS), with the SUS score calculated as the respective outcome measure [24]. Clinical relevance was determined by the number of detected adverse events, specifically neutropenia and QTc prolongations.

### DHHC Hardware

The DHHC consisted of the following components: (1) Apple Watch Series 6 (Apple Inc., Cupertino, CA, United States) for ECG measurements, (2) HemoCue WBC DIFF Analyzer (HemoCue AB, Ängelholm, Sweden) for WBC counts, (3) iPhone SE (Apple Inc.) for QoL questionnaire completion and photo documentation, (4) mobile Wi-Fi router TP-Link M7350-4G (TP-Link Corporation Limited, Düsseldorf, Germany), and (5) Raspberry Pi 4 Model B (Raspberry Pi, Cambridge, United Kingdom).

Two cases were designed to enable safe and easy transport and handling of the devices (Figure 1B-F). Case 01 (Figure 1B-D; Fa. Karl Lettenbauer, Erlangen, Germany) featured a plastic base plate to accommodate the HemoCue WBC DIFF Analyzer, Raspberry Pi, power cable, and socket strip (installed under the upper mount with a 14412‐02 detachable partition protected against tampering), along with all device cables. The iPhone, Apple Watch, and TP-Link were integrated into a raised platform. Case 02 (Figure 1E-F) consisted of a case from MyCaseBuilder.eu (FOAM Studio, the Netherlands) with a custom Pro-Cell interior and Prolife Soft-Cell foam lid. For both cases, the devices could be charged, and WBC measurements could be taken without the devices being removed from the cases.

### SMILER.one App and DHHC Software

The custom study app (SMILER.one) was developed by REFINIO GmbH, based on their REFINIO ONE architecture. REFINIO GmbH is a German company specializing in custom software for secure data collection. The software was programmed in TypeScript on NodeJS and had platform abstractions for internet browsers and Linux. All ONE instances of a person formed a federation called the Internet of Me (IoM), where identities, connections, settings, and content could be distributed, ensuring that devices only needed to be registered once.

Data storage in ONE was based on HTML files containing microdata objects, which were stored in individual files within the file system or in the IndexedDB of the browser or WKWebView on mobile devices. The objects were named according to the hash of their content and referenced through their name in parent objects. Data transmission in ONE was facilitated through WebSocket services provided by a commServer, which established connections between devices. Data sharing was based on subtree sharing and conflict-free replicated data types, with encryption occurring at the individual instance level.

The DHHC integrated several REFINIO ONE software components, including the Web Server, SMILER.one Mobile, SMILER.one Pi, SMILER.one Headless, and the SMILER.one representational state transfer application programming interface (REST API). The Web Server was installed and configured with HTTPD software (nginx) to deliver the SMILER.one Progressive Web App and mobile content. SMILER.one Mobile managed patient data in a WebView (IndexedDB) on an iPhone and synchronized it with the clinic’s data (SMILER.one Headless) and the patients’ IoM instances. It also imported and synchronized ECG data from the Apple HealthKit on patients’ iPhones. SMILER.one Pi was installed on the Raspberry Pi, importing WBC data from the HemoCue device. Within the patients’ IoM, it acted as a headless replication of the patients’ complete dataset. SMILER.one Headless mirrored all settings and storage operations of SMILER.one Mobile and SMILER.one Pi while incorporating the SMILER.one REST API, which provided pseudonymized patient data to the clinic’s SQL server (Figure S1 in Multimedia Appendix 1).

The SMILER.one app served as a user interface for study participants. The app featured a registration or login mechanism with password encryption to ensure restricted private access. Within the app, the “My Tasks” screen allowed participants to complete visits (questionnaires, photo documentation, ECG measurements, and WBC counts) and provided an overview of upcoming tasks (Figure 1G). In the “Data Archive,” completed data were stored and could be viewed by the participants (Figure 1H). The “Blood Count Chart” displayed a chart of WBC readings from the HemoCue device (Figure 1I). Patients also had the option to enter additional data beyond the scheduled remote visits under “Voluntary Data Entry.”

### Data Management

Data from the DHHC was stored in a dedicated relational database on an SQL server. The data transfer from the REFINIO REST API to the SQL database was facilitated via the JSON data format. Parsing of the JSON-formatted data and transformation into a relational tabular format were performed within the database itself. QTc times were calculated from the transmitted ECG curves using the Fridericia formula by a physician (PK). Based on the QTc times and measured neutrophil concentrations, the severity of QTc prolongations and neutropenia was graded according to the Common Terminology Criteria for Adverse Events version 5.0. Clinical data were collected by trained staff and documented in an electronic case report form. Data monitoring was conducted using automated plausibility checks and on-site monitoring. These data included patient and tumor characteristics, as well as details on treatment approaches.

### Statistical Analysis

Sample size calculations for this feasibility study were based on the assumption that 70% of ECG measurements and 65% of WBC counts would be successful (two 1-sided exact binomial tests with a significance level of ɑ=2.5%). A total of 212 ECG measurements and 237 blood measurements were required to demonstrate this with a power of 90%, which corresponded to 80 patients, each with three ECG measurements and three WBC count measurements.

The majority of the presented statistics are descriptive. Categorical variables are reported as counts and percentages, while continuous variables are presented as mean (SD). Missing values were omitted from analyses. A 2-sided Wilcoxon rank sum test was used for statistical comparisons, with *P*≤.05 considered statistically significant. Data are presented as box plots, displaying the median, IQRs, and whiskers representing the 5th and 95th percentiles. Statistical analyses were performed using R (version 4.2.1; The R Foundation) or SPSS Statistics (version 29.0.1.0; IBM Corp). Likert plots were generated using the R library “Likert” (version 1.3.5). The distribution of Likert scale questionnaire scores ranged from 1=I fully agree to 5=I do not agree at all.

## Results

### Feasibility

#### Recruitment

**Figure 2.**
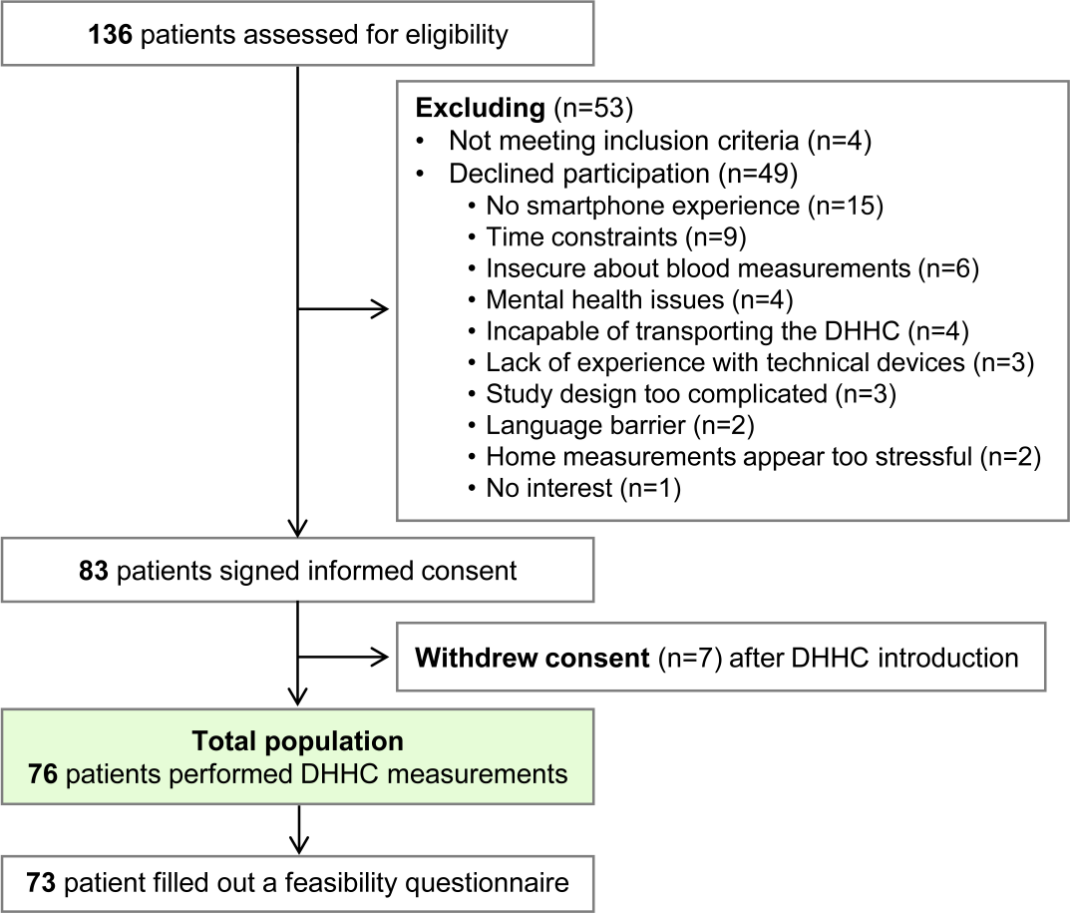
Patient flowchart. This flowchart illustrates the progression of patients throughout the study, including screening, enrollment, and final study participants. DHHC: digital home health care center.

Between October 2021 and December 2022, 136 patients with breast cancer were screened for eligibility. Of the 132 eligible patients, 49 patients declined participation. The most common reasons for nonparticipation were lack of smartphone experience (n=15) and lack of time to complete study procedures (n=9; Figure 2). Of the 83 patients who provided informed written consent, 7 patients withdrew their consent after being introduced to the DHHC (Figure 2), resulting in 76 patients who participated in the SMILER study. Of these 76 patients, 73 (96%) completed the questionnaire on acceptance, success rate, and feasibility of the home-based procedures at study completion.

#### Patient Characteristics

Participants had a mean age of 58.9 (SD 9.74) years (Table 1). The majority of participants received CDK4/6i for advanced or metastatic disease (54/76, 71%). Abemaciclib was administered to 49% (37/76) of participants, while 39% (30/76) and 12% (9/76) of participants received ribociclib and palbociclib, respectively. Additional baseline patient characteristics are listed in Table 1. Case 01 was assigned to 33% (25/76) of participants, while Case 02 was provided to 67% (51/76) of participants.

**Table 1.** Baseline characteristics of study participants.

Characteristics	Study participants (n=76)	
Age (years), mean (SD)	58.9 (9.74)	
BMI^a^ (kg/m^2^), mean (SD)	25.7 (4.65)	
ECOG^b^ index^c^, n (%)		
0	68 (91)	
1	6 (8)	
2	0 (0)	
3	1 (1)	
Grading^d^, n (%)		
G1	7 (10)	
G2	40 (54)	
G3	27 (36)	
ER^e^, n (%)		
ER+	76 (100)	
ER–	0 (0)	
PR^f^, n (%)		
PR+	62 (82)	
PR–	14 (18)	
HER2/neu status, n (%)		
HER2+	6 (8)	
HER2–	70 (92)	
CDK4/6^h^ inhibitor, n (%)		
Abemaciclib	37 (49)	
Palbociclib	9 (12)	
Ribociclib	30 (39)	
Endocrine therapy combination partner, n (%)		
Aromatase inhibitor	54 (71)	
Fulvestrant	16 (21)	
Other	2 (3)	
Metastasis, n (%)		
M0	22 (29)	
M1	54 (71)	
Line of therapy, n (%)		
1st line	42 (78)	
2nd line	7 (13)	
3rd line or higher	5 (9)	
Highest degree of education^g^, n (%)		
No degree	0 (0)	
General secondary school	12 (17)	
Intermediate secondary school	14 (20)	
University of Applied Science entrance certificate	5 (7)	
University entrance certificate	4 (5)	
Vocational training	22 (31)	
Bachelor’s degree	0 (0)	
Master’s degree or higher	14(20)	
SMILER case, n (%)		
Case 01	25 (33)	
Case 02	51 (67)	

a Missing: n=5.

b ECOG: Eastern Cooperative Oncology Group.

c Missing: n=1.

d Missing: n=2.

e ER: estrogen receptor.

f PR: progesterone receptor.

g Missing: n=5.

h CDK4/6: cyclin-dependent kinase 4 and 6.

### Adherence

**Figure 3.**
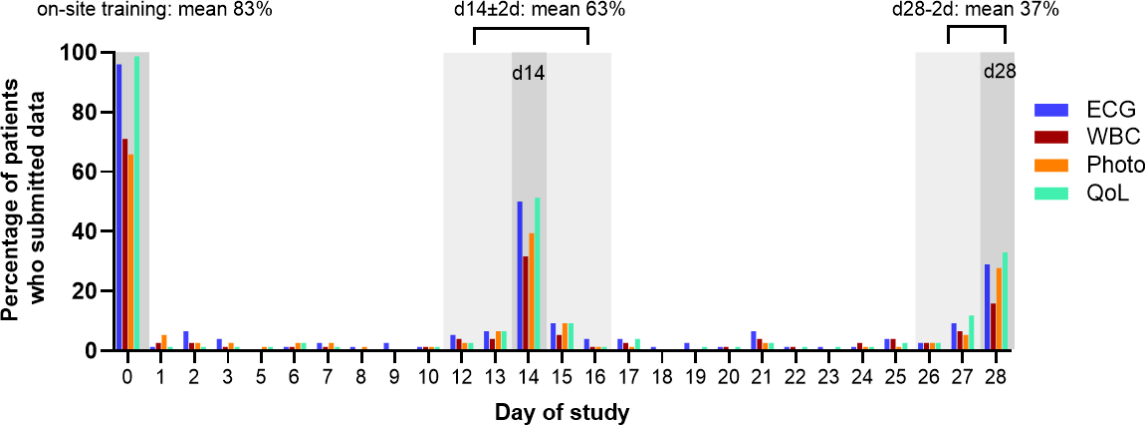
Patients who submitted data during the study. The percentage of patients who submitted data via the digital home-based health care center is presented for various measurements, including ECG, WBC counts, photo documentation, and QoL questionnaires. The bars indicate the data submission rates over the study duration, with dark gray sections representing planned study visits (day 14, d14; and day 28, d28) and light gray sections representing the days around the study visits (d14±2, d28-2). The mean percentage represents the average data submission rate for each study visit. ECG: electrocardiogram; QoL: quality of life; WBC: white blood cell.

Study-relevant measurements were scheduled at study inclusion (introductory on-site training), day 14 (d14—home-based), and day 28 (d28—home-based). The average adherence was 63% at d14±2d and 37% at d28-2d (Figure 3). Among individual remote measurements, ECGs were recorded most frequently (d14±2d: 57/76, 75%; d28-2d: 31/76, 41%), followed by QoL questionnaires (d14±2d: 54/76, 71%; d28-2d: 36/76, 47%).

The most common patient-reported reasons for missing scheduled tasks were device malfunction (ECG or Apple Watch: 11/36, 31%; WBC or HemoCue: 16/43, 37%; photo documentation: 5/31, 16%), handling issues (ECG or Apple Watch: 11/36, 31%; WBC or HemoCue: 12/43, 28%; photo documentation: 2/31, 6%), and time constraints (ECG or Apple Watch: 10/36, 28%; WBC or HemoCue: 10/43, 23%; photo documentation: 11/31, 35%; Table 2). Among the patient-reported reasons listed as “other reasons” for failed adherence were “measurements were not performed in time,” “unsure about the handling of the devices,” “no reception,” “mentally too stressed,” or “sickness.” Among these, “unsure about the handling of the device” was the most common other reason for missed WBC measurements (5/13, 38%).

**Table 2.** Patient-reported reasons for not being able to perform scheduled home-based measurements (d14 and d28le)^a^.

	ECG^b^ (n=36), n (%)	WBC^c^ (n=43), n (%)	Photo documentation (n=31), n (%)
Device would not function	11 (31)	16 (37)	5 (16)
Measurement could not be performed properly	11 (31)	12 (28)	2 (6)
Battery was empty	3 (8)	N/A^d^	3 (10)
Safety concerns	N/A	2 (5)	1 (3)
No time	10 (28)	10 (23)	11 (35)
No interest	N/A	N/A	1 (3)
Other reasons	15 (42)	13 (30)	12 (39)

a Patients could indicate their reason for nonadherence to the questionnaire upon completion of the study. Participants could choose from predefined reasons, with multiple answers possible. Responses were only requested from study participants who indicated that they were unable to perform all scheduled measurements. When selecting “other reasons,” patients could provide additional details in a blank text field.

b ECG: electrocardiography.

c WBC: white blood cell count.

d N/A: not applicable.

### Success Rates

Throughout the 28-day study period, data transfer was expected from three ECG measurements, three WBC measurements, three QoL questionnaires, and six photo documentations (each ankle per time point) per patient. The mean number of successfully transferred data per patient during the study period was 3.04 (SD 1.9), ECGs was 2.14 (SD 1.1), WBC measurements was 2.5 (SD 1.1), QoL questionnaires and photo documentations was 4.4 (SD 3.4; Table S1 in Multimedia Appendix 1).

Study participants indicated how many times a measurement had to be repeated before it was successfully completed in the end-of-study questionnaire. The highest number of repetitions was required for WBC measurements, with an average of 1.07 (SD 1.0) additional measurements per patient (Table S1 in Multimedia Appendix 1).

### Identified Problems

Several issues may have affected both study adherence and success rates. The incidence of any type of DHHC malfunction (ie, problems with either WBC or ECG measurements) was 24% (18/76) during first use (initial introductory training) and 43% (33/76) during remote measurements (as reported in the end-of-study questionnaire).

For WBC measurements, handling problems with the HemoCue microcuvettes requiring repeat WBC measurements were observed in 8 (out of 76, 10%) patients during on-site training. Of these, 2 (25%) patients also reported handling issues with WBC measurements during remote monitoring. Patients who experienced handling problems during initial training (8/76) and those who reported handling issues as a reason for nonadherence to remote monitoring visits (12/76) appeared to be older than those without handling issues (initial training: handling issues 59.4, SD 9.6 y vs no handling issues 55.0, SD 10.3 y; *P*=.30; remote monitoring: handling issues 63.3, SD 7.6 y vs no handling issues 58.1, SD 9.9 y; *P*=.02).

At initial training, technical problems with the HemoCue WBC DIFF IEC 61010 system (failure to turn on or instant error messaging) prevented measurements in 4 (out of 76, 5%) patients and data transfer from the HemoCue WBC DIFF IEC 61010 system to the SMILER.one app and SQL server in 11 (out of 76, 14%) patients. In two of these cases (2/11, 18%), a lack of an internet connection was reported.

Defective data transfer occurred more often with Case 02 than Case 01 (8/11, 73% vs 3/11, 27%) and in two instances, the same Case 02 DHHC experienced defective data transfer. However, the case type was not statistically associated with defective data transfer (*P*=.80). Among 3 (out of 11, 27%) DHHCs with defective data transfer during on-site training, study participants also reported an inability to perform remote measurements due to device malfunction.

For ECG measurements, defective data transfer was observed in 3 (out of 76, 4%) individual DHHCs during on-site training, and 1 (out of 3, 33%) patient also reported subsequent technical issues with the Apple Watch during remote monitoring. Additionally, 4% (3/76) of patients reported being unable to use the SMILER.one app due to a lack of mobile network coverage.

### User Perception of Feasibility

**Figure 4.**
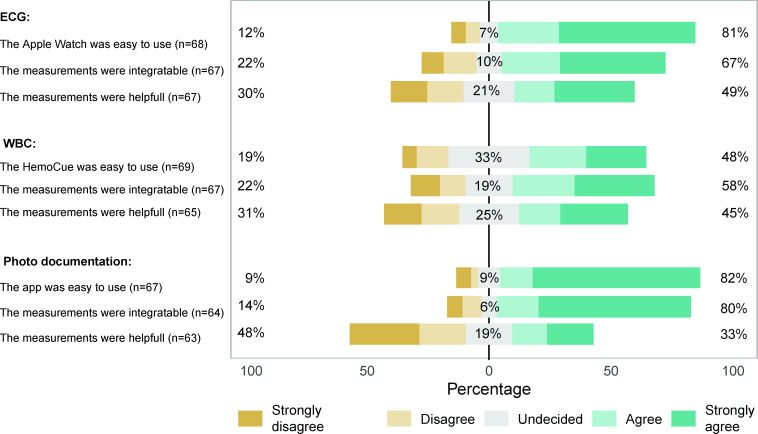
User perception of the home-based health care system’s feasibility. The Likert scale illustrates responses from a user perception questionnaire regarding the Apple Watch ECG, HemoCue WBC measurement, and photo documentation via the SMILER.one app. ECG: electrocardiogram; WBC: white blood cell.

The feasibility of home measurements was assessed at study completion. While most patients found photo documentation with the SMILER.one app (55/67, 82%) and ECG measurements with the Apple Watch (55/68, 81%) easy to use, only 48% (33/69) of patients found the HemoCue System easy to use. Good integration into daily life was most commonly reported for photo documentation (51/64, 80%), followed by ECG (45/67, 67%) and WBC measurements (39/67, 58%). ECG and WBC measurements were seen as helpful by 49% (33/67) and 45% (29/65) of patients, respectively, while only 33% (21/63) of participants found photo documentation helpful (Figure 4).

### Acceptance

**Figure 5.**
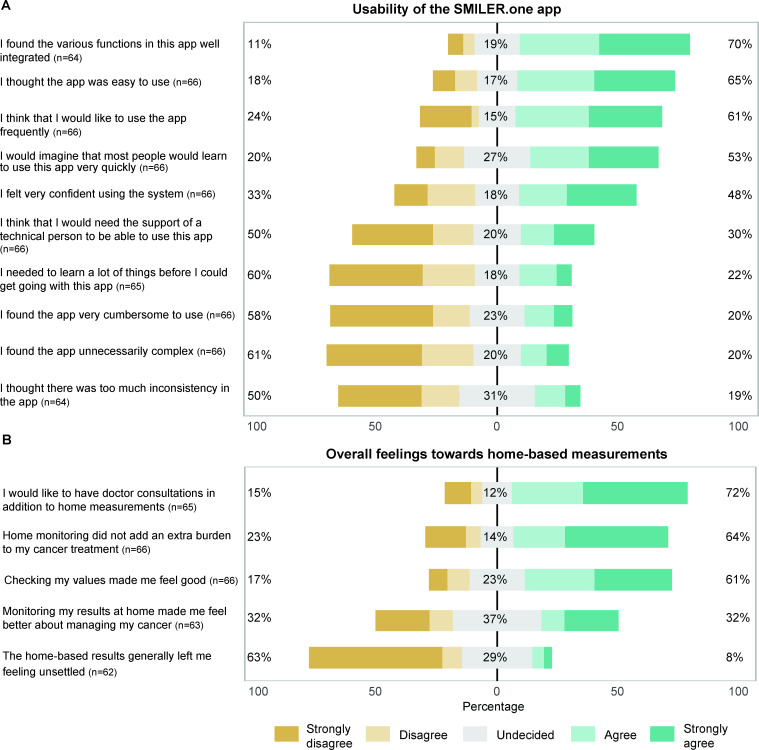
User perception and acceptance of the SMILER.one app and the home-based health care system. (A) The Likert scale illustrates responses from a user perception and acceptance questionnaire of the SMILER.one app and (B) the complete home-based health care system.

User acceptance of the SMILER.one app and the perception of the DHHC and its future use were assessed through the end-of-study questionnaire. For the SMILER.one app, 70% (45/64) of participants agreed that the technical features of the app were well-integrated, 65% (43/66) of participants found the app easy to use, and 61% (40/66) of participants could imagine using the app regularly. However, 30% (20/66) of participants reported that they would need technical support to use the app (Figure 5A). The corresponding SUS score for the SMILER.one app was 65.2. Regarding the DHHC, 61% (40/66) of participants felt comfortable with self-monitoring, and 64% (42/66) of participants did not consider home-based measurements to be an additional burden. However, 72% (47/65) of participants stated that they would like to have consultations with a doctor in addition to the DHHC measurements (Figure 5B).

When asked about their intention to use, 79% (54/68) of participants expressed a willingness to use home measurements as part of their future cancer care. Additionally, 79% (54/68) of participants were willing to collect data for research purposes at home using the SMILER.one app. Participants who were unwilling or reluctant to integrate the DHHC and remote data collection into future cancer care reported that they would need help interpreting results (9/17, 53%), were concerned about the time required for data collection (11/23, 48%), and would experience difficulties or stress related to the technology (home-based measurements: 3/17, 18%; collection and sharing of data with the SMILER.one app: 7/23, 30%; Table S2 in Multimedia Appendix 1).

### Clinical Relevance

Transferring routine monitoring from the clinical setting to the at-home environment requires the evaluation of WBC counts and ECG values. WBC monitoring is necessary for all CDK4/6i therapies, whereas ECG monitoring, specifically assessing QTc intervals, is required only for ribociclib treatment.

**Figure 6.**
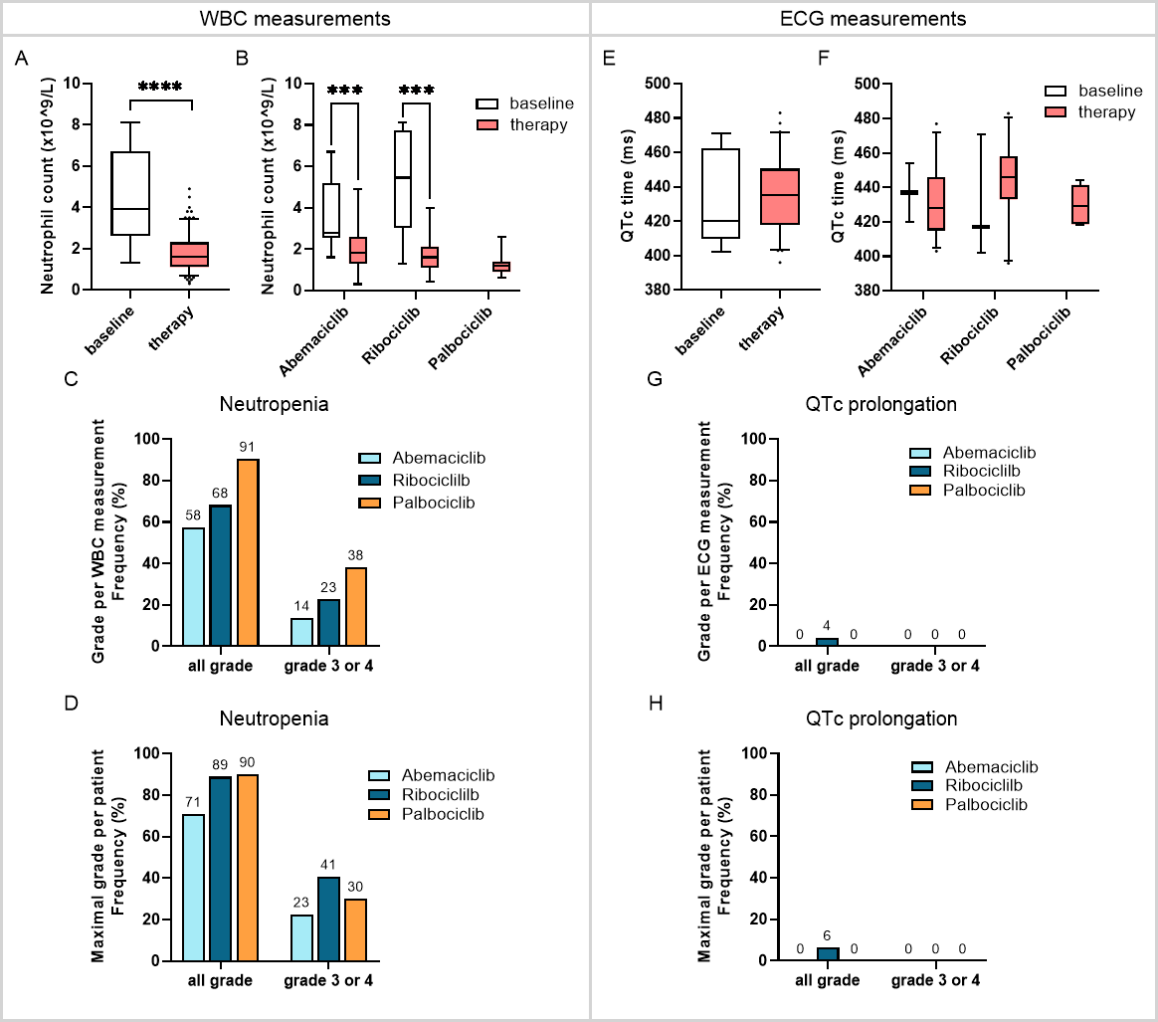
Detection of adverse events with the digital home health care center. Neutrophil counts were measured with the HemoCue system. (A) Neutrophil count before starting CDK4/6i therapy (n=15) and under therapy (>5 days; n=167) of all combined WBC measurements and (B) per CDK4/6i (baseline: abemaciclib, n=9; ribociclib, n=6; under therapy: abemaciclib, n=80; ribociclib, n=66; palbociclib, n=21). Neutropenia was graded according to CTCAE version 5.0. Frequency of neutropenia under CDK4/6i therapy as (C) the grade of each individual WBC measurement (abemaciclib, n=80; ribociclib, n=66; palbociclib, n=21) and (D) the maximal observed grade per patient (abemaciclib, n=31; ribociclib, n=27; palbociclib, n=10). QTc times were calculated from ECGs measurements with the Apple Watch. (E) QTc time before starting CDK4/6i therapy (n=5) and under therapy (>5 days; n=61) of all combined ECG measurements and (F) per CDK4/6i (baseline: abemaciclib, n=2; ribociclib, n=3; under therapy: abemaciclib, n=33; ribociclib, n=23; palbociclib n=5). QTc prolongation was graded according to CTCAE version 5.0. Frequency of QTc prolongation under CDK4/6i therapy as (G) the grade of each individual QTc measurement (abemaciclib, n=33; ribociclib, n=23; palbociclib n=5) and (H) the maximal grade per patient (abemaciclib, n=21; ribociclib, n=16; palbociclib n=5). ***P≤.001, ****P<.001; Wilcoxon rank sum test. CDK4/6i: cyclin-dependent kinase 4/6 inhibitor; CTCAE: Common Terminology Criteria for Adverse Events; ECG: electrocardiogram; QTc: corrected QT interval; WBC: white blood cell.

Compared with patients initiating CDK4/6i therapy, those who had been on treatment for more than five days had lower neutrophil counts (mean 4.3, SD 2.2, ×10^9^/L vs mean 1.8, SD 0.8, ×10^9^/L; *P*<.001; Figure 6A), regardless of the specific CDK4/6i received (Figure 6B). Mild neutropenia (grade 2) was present in 27% (45/167) of WBC measurements, while severe neutropenia (grade 3 or 4) was detected in 20% (34/167) of WBC measurements (Table S3 in Multimedia Appendix 1). At the patient level, this corresponded to 32% (22/68) of patients experiencing mild neutropenia and 31% (21/68) presenting with severe neutropenia under CDK4/6i therapy, as detected by the DHHC (Table S4 in Multimedia Appendix 1). Regarding individual CDK4/6i therapies, grade 3 or 4 neutropenia was observed in 23% (7/31) of patients receiving abemaciclib, 41% (11/27) of those on ribociclib, and 30% (3/10) of those on palbociclib (Figure 6C-D and Table S5 in Multimedia Appendix 1).

QTc time remained stable under CDK4/6i therapy (Figure 6e,f). Only one measurement indicated QTc prolongation, corresponding to 2% (1/61) of all quantifiable QTc times from ECG measurements and 2% (1/42) of all patients (Tables S5 and S6 in Multimedia Appendix 1). The affected patient was receiving ribociclib (Figure 6g.h and Tables S5 and S6 in Multimedia Appendix 1).

## Discussion

### Principal Findings

The SMILER study assessed the feasibility and acceptance of a DHHC system for remote monitoring of side effects in patients with breast cancer receiving CDK4/6i therapy. Adherence to remote study visits was moderate and declined over time, with patients who were unable to perform measurements reporting time constraints and handling issues with the devices. Correspondingly, the transfer of remotely collected data was lower than expected, and patients reported needing to perform repeat measurements. Self-monitoring of ECGs and side effect photo documentation was considered easy to use and easily integrable into daily life, whereas WBC measurements were generally found to be more challenging. Nevertheless, the majority of participants felt positive about self-monitoring and expressed a willingness to incorporate home-based measurements into their future cancer care. Additionally, home-based ECG and WBC measurements were effective in detecting QTc time prolongations and neutropenia, demonstrating the clinical relevance of the DHHC.

### Comparison to Prior Work

Our findings contribute to the emerging field of digital health and remote monitoring, highlighting the potential of such interventions in improving patient care and outcomes. Patients receiving oral CDK4/6i therapy can take their anticancer medication at home; however, clinic visits remain necessary to monitor potential side effects [25-27]. Severe neutropenia (grade 3 or 4) is commonly observed under CDK4/6i therapy (ribociclib: 57%‐62% of patients in randomized controlled trials [RCTs] and 15%‐69% of patients in real-world studies [3,28-33]; abemaciclib: ~20% of patients in RCTs and 2%‐24% in real-world studies [9,31,33,34]; palbociclib: 62%‐70% of patients in RCTs and 60%‐63% in real-world studies [5,33,35-37]). Home-based WBC measurements by patients with breast cancer detected severe neutropenia in 41% of patients on ribociclib, 23% of patients on abemaciclib, and 30% of patients on palbociclib, aligning with real-world data for abemaciclib and ribociclib. Severe neutropenia under palbociclib may be underestimated due to the small number of patients receiving palbociclib in this study population. Nevertheless, these findings support the clinical relevance of home-based WBC monitoring with the DHHC.

QTc prolongation is a specific potential side effect of ribociclib therapy and has been reported in 2%‐5% of patients in the different RCTs [2-4]. In this study, prolonged QTc times were detected in one patient, corresponding to 6% (1/76 patients) of those receiving ribociclib therapy, which is in line with RCT findings [2-4]. Patients receiving abemaciclib or palbociclib did not develop QTc prolongations during the SMILER study.

Further research is needed to evaluate the clinical and psychological effects of home-based monitoring. A recent randomized trial involving patients with breast cancer receiving palbociclib therapy found that patients using an eHealth app (CANKADO PRO-React) experienced a longer time to QoL deterioration compared with those who did not use the app [19]. This study further highlights the clinical potential of digital interventions in improving patient outcomes and well-being. Notably, mobile health apps developed by health care professionals appear to have the highest overall quality [38], emphasizing the need to integrate clinical expertise with innovative digital approaches.

User perception and acceptance of the DHHC were generally positive. Both the ECG measurements with the Apple Watch and the photo documentation with the SMILER.one app were considered easy to use. The integration of the measurements into daily life was perceived favorably. However, concerns about result interpretation, time-consuming data collection, and technological difficulties were reported as barriers to engagement. The SUS score also indicated moderate usability. These findings emphasize the importance of providing adequate support and guidance to patients in using and interpreting the collected data. Additionally, the preference for consultations with health care professionals highlights the need to integrate home-based monitoring with clinical care and medical expertise. For future developments, integrating a communication tool into the provided app could be a suitable solution. Implementing a direct, automated digital feedback system based on home-based measurements could offer patients a more immediate and informed understanding of their health status, potentially reducing the need for frequent consultations with doctors. Interestingly, both using medical apps for medical questions and consulting Google appear to result in comparable adverse emotional and behavioral effects associated with cyberchondria [39].

Virtual and remote trials often have high dropout rates or low adherence to visits [40-42]. In the SMILER trial, adherence rates varied across different measurements, with the highest adherence for QoL questionnaires and lower adherence for WBC and ECG measurements. Additionally, adherence to the scheduled remote visits gradually declined over the course of the study, with 63% on d14±2d and 37% on d28-d2. Notably, several patients performed home-based measurements before or after the scheduled visits. Some patients also reported being unable to complete the final measurement due to the automatic deactivation of the SMILER.one app at the end of the study (day 28), suggesting that adherence rates may be underestimated.

The most common reason for missed scheduled measurements was device malfunction, with both technical and handling issues reported. Notably, the incidence of any type of malfunction during the first on-site introductory training was 24% (18/76 participants), and patients reported problems with 43% (33/76) of the DHHCs during remote monitoring. Not all patients with partially malfunctioning DHHC devices at the introductory training reported subsequent issues during remote monitoring, which may indicate that these devices were functional again during remote measurements or that the patient-reported failure rate was incomplete. Technical problems with the HemoCue device and the lack of an internet connection may partly explain DHHC malfunctions. Although the underlying cause of defective data transfer could not be definitively determined, server issues and complications with the Raspberry Pi software may have contributed. It is possible that unplugging the DHHC between remote measurements affected the Raspberry Pi and its software, which facilitates data transfer from the HemoCue device to the SMILER.one app. While there was no clear correlation between case design and the reported issues, data transfer problems occurred at varying rates across the different cases.

Handling problems with the HemoCue microcuvettes, such as an incomplete filling or air bubbles in the sample, was observed in approximately 10% (8/76) of patients during their first on-site WBC measurement. This is comparable to findings from another study that evaluated the feasibility of WBC self-testing with the HemoCue system in patients with cancer [43]. Additionally, handling issues during WBC measurements were associated with older age, highlighting the need for either a more intensive training session or remote assistance from a trained nurse, particularly for older patients. In general, tailoring support to specific patient needs may be essential to ensure successful implementation alongside a robust and reliable technological infrastructure.

Recruitment and patient characteristics also play a crucial role in the success of a study. In the SMILER study, only 76 out of the 136 screened women ultimately participated. Reasons for nonparticipation included lack of smartphone experience, time constraints, and concerns about performing WBC count independently. This underscores the importance of understanding the target population and their readiness to engage with digital health interventions. It is crucial to design digital interventions in a way that ensures a lack of digital literacy does not contribute to health inequality [44,45].

### Strengths

This study demonstrates several strengths in the implementation and evaluation of a DHHC system for patients receiving CDK4/6i therapy. First, it confirms the clinical relevance of remote monitoring by detecting known side effects, such as neutropenia and QTc prolongations, consistent with the established side effect profiles of these therapies. Although not designed to detect differences in the incidence of neutropenia or ECG changes between CDK4/6i, commonly known differences were observed [46]. Patients treated with ribociclib and palbociclib experienced neutropenia more frequently than those treated with abemaciclib. The only patient who had a QTc prolongation was receiving ribociclib.

Second, the study successfully integrated multiple technologies, including WBC measurement systems, smartwatches, and smartphones, into a comprehensive system tailored to the patient’s needs. The high level of patient acceptance, with 79% (54/68) of participants expressing a willingness to use such systems in their future care, highlights the feasibility of this patient-centered approach. Moreover, the developed DHHC system and study findings underscore the potential of remote monitoring to reduce disparities in cancer care, particularly for patients in rural areas.

### Limitations

This study has several limitations. First, the small sample size, specific patient population, and potential selection bias due to nonparticipation may limit the generalizability of the results. Second, adherence rates were suboptimal, and technical issues were encountered, highlighting challenges in implementing and maintaining home-based health care systems. Further optimization of the remote monitoring system is needed to improve usability. Additionally, a detailed analysis of the reliability of the DHHC could not be performed due to insufficient data. Third, self-reporting bias may have influenced the reported perceptions and experiences of the participants. Fourth, the short study duration limits our understanding of the long-term feasibility and acceptance of remote monitoring interventions. Fifth, the study lacked a control arm with patients receiving traditional nursing care, making direct comparisons with standard care models difficult. Sixth, as this was a pilot study, we did not predefine specific thresholds for the evaluated outcomes, such as adherence, usability, and feasibility, which limits our ability to determine whether the observed rates met predefined success criteria. Finally, this study did not include an analysis of the economic feasibility of the DHHC.

### Future Directions

Based on the outcomes of this first feasibility study, several areas for improvement were identified. The technical issues encountered may be related to the DHHC devices, internet connection, server, Raspberry Pi software, and other factors that need to be addressed. Furthermore, longer-term randomized controlled studies are needed to better assess patient compliance, system reliability, and economic impact.

### Conclusions

The findings of this study demonstrate that implementing remote monitoring of side effects in the care of patients with breast cancer undergoing CDK4/6i therapy is feasible. Patients with breast cancer generally accepted the idea of remote monitoring, which successfully identified clinically relevant side effects. Future studies with an improved system are required to further evaluate the potential clinical, socioeconomic, and individual benefits of this approach.

## Supplementary material

10.2196/64083 Multimedia Appendix 1 Combination of supplementary materials.

## Abbreviations:

CDK4/6i: cyclin-dependent kinase 4/6 inhibitor
DHHC: digital home-based health care center
ECG: electrocardiogram
IoM: Internet of Me
QoL: quality of life
QTc: corrected QT interval
RCT: randomized controlled trial
REST API: representational state transfer application programming interface
SUS: System Usability Scale
WBC: white blood cell
