# Supplementary material for: A Digital Home-Based Health Care Center for Remote Monitoring of Side Effects During Breast Cancer Therapy: Prospective, Single-Arm, Monocentric Feasibility Study
Source: JMIR Cancer. 2025 May 2;11:e64083. doi: 10.2196/64083 (PMC12064077; doi:10.2196/64083)
Supplement: Multimedia Appendix 1 [file cancer-v11-e64083-s001.docx]

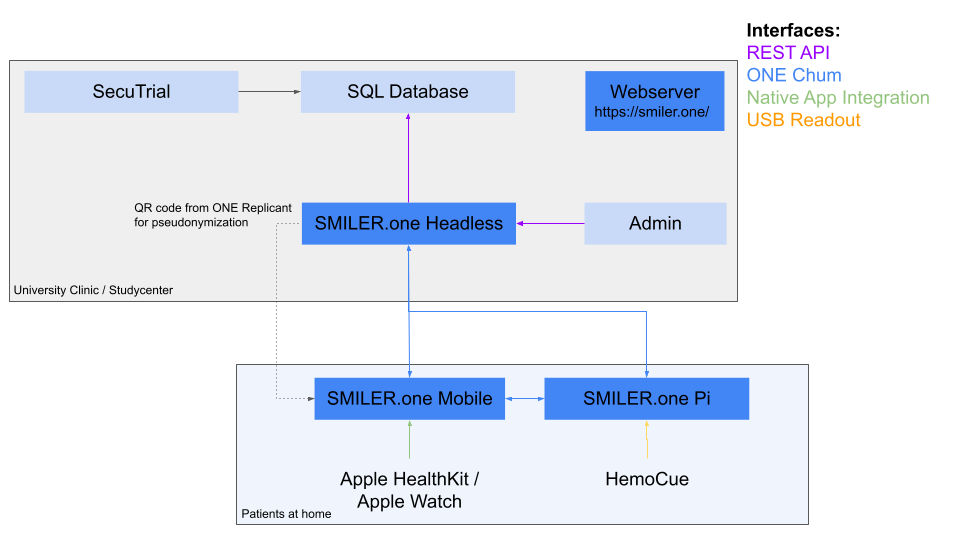


**Figure S1**: SMILER software set-up.

**Table S1:** Success rate of home-based measurements.

|  | ECG  Mean (±SD) | WBC  Mean (SD) | Photo documentation  Mean (SD) | QoL  Mean (SD) |
| --- | --- | --- | --- | --- |
| Average number of successfully transferred data to the central data server, per patient (DT)^1^ | 3.0 (±1.9) | 2.1 (±1.1) | 4.4 (±3.4) | 2.5 (±1.1) |
| Average required repetitions to achieve a successful measurement, per patient (SR)² | 0.8 (±0.9) | 1.0 (±1.0) | 0.79 (±1.0) | NA |

[ECG: Electrocardiography; WBC: White blood cell count; SD: standard deviation SR: self-reported using questionnaire, DT: data successfully transferred to central server, QoL: patient-reported outcome questionnaire EQ5D3L] ^1^ During the study period; ² 0 representing no repletion necessary to achieve successful measurement and ≥1 representing the numbers of additional measurements

Table S2: Reasons why patients are unwilling to perform furture home-based measurements

| **Reasons why patients are hesitant to perform home-based measurements as part of their cancer treatment (N=17) -** N (%) | |
| --- | --- |
| Help required with result interpretation | 9 (53) |
| Technology is too difficult/causes too much stress | 3 (18) |
| Do not constantly want to be confronted with the disease | 2 (12) |
| Takes too much time | 2 (12) |
| Concerns regarding data protection | 2 (12) |
| No interest | 1 (6) |
| **Reasons why patients are hesitant to collect and share home-based data via the SMILER.ONE app (N=23) -** N (%) | |
| Technology is too difficult/causes too much stress | 7 (30) |
| Takes too much time | 11 (48) |
| Concerns regarding data protection | 2 (9) |
| Home measurements are not always practical | 2 (9) |
| Home measurements make me feel insecure | 2 (9) |
| No interest | 1 (4) |

Table S3: Neutropenia severity grading of all combined individual white blood cell measurements made with the digital and mobile home healthcare center. Measurements after at least 5 days of CDK4/6 inhibitor therapy (CDK4/6i) were included in analysis. Multiple measurements per patient were possible. Neutropenia was graded according to Common Terminology Criteria for Adverse Events (CTCAE) version 5.0.

| **Neutropenia** | **All CDK4/6i**  **(N=167)**  **N (%)** | **Abemaciclib**  **(N=80)**  **N (%)** | **Ribociclib**  **(N=66)**  **N (%)** | **Palbociclib**  **(N=21)**  **N (%)** |
| --- | --- | --- | --- | --- |
| Grade 1 | 31 (18) | 16 (20) | 13 (20) | 2 (9) |
| Grade 2 | 45 (27) | 19 (24) | 17 (26) | 9 (43) |
| Grade 3 | 30 (18) | 9 (11) | 13 (20) | 8 (38) |
| Grade 4 | 4 (2) | 2 (2) | 2 (3) | 0 (0) |

Table S4: Neutropenia grading per patient. White blood cell measurements were performed with the digital and mobile home healthcare center. The highest grade per patient under CDK4/6 inhibitor (CDK4/6i) therapy (> 5 days of therapy) was used for calculations. Neutropenia was graded according to Common Terminology Criteria for Adverse Events (CTCAE) version 5.0.

| **Neutropenia** | **All CDK4/6i**  **(N=68)**  **N (%)** | **Abemaciclib**  **(N=31)**  **N (%)** | **Ribociclib**  **(N=27)**  **N (%)** | **Palbociclib**  **(N=10)**  **N (%)** |
| --- | --- | --- | --- | --- |
| Grade 1 | 12 (18) | 6 (19) | 6 (22) | 0 (0) |
| Grade 2 | 22 (32) | 9 (29) | 7 (26) | 6 (60) |
| Grade 3 | 17 (25) | 5 (16) | 9 (33) | 3 (30) |
| Grade 4 | 4 (6) | 2 (6) | 2 (7) | 0 (0) |

Table S5: QTc prolongation severity grading of all quantifiable QTc times from all combined ECGs made with the digital and mobile home healthcare center. Measurements after at least 5 days of CDK4/6 (CDK4/6i) inhibitor therapy were included in analysis. Multiple measurements per patient were possible. QTc prolongation was graded according to Common Terminology Criteria for Adverse Events (CTCAE) version 5.0.

| **QTc prolongation** | **All CDK4/6i**  **(N=61)**  **N (%)** | **Abemaciclib**  **(N=33)**  **N (%)** | **Ribociclib**  **(N=23)**  **N (%)** | **Palbociclib**  **(N=5)**  **N (%)** |
| --- | --- | --- | --- | --- |
| Grade 1 | 0 (0) | 0 (0) | 0 (0) | 0 (0) |
| Grade 2 | 1 (2) | 0 (0) | 1 (4) | 0 (0) |
| Grade 3 | 0 (0) | 0 (0) | 0 (0) | 0 (0) |
| Grade 4 | 0 (0) | 0 (0) | 0 (0) | 0 (0) |

Table S6: QTc prolongation grading per patient. ECG measurements were performed with the digital and mobile home healthcare center and the QTc time was quantified. The highest grade per patient under CDK4/6 inhibitor (CDK4/6i) therapy (> 5 days of therapy) was used for calculations. QTc time was graded according to Common Terminology Criteria for Adverse Events (CTCAE) version 5.0.

| **QTc prolongation** | **All CDK4/6i**  **(N=42)**  **N (%)** | **Abemaciclib**  **(N=21)**  **N (%)** | **Ribociclib**  **(N=16)**  **N (%)** | **Palbociclib**  **(N=5)**  **N (%)** |
| --- | --- | --- | --- | --- |
| Grade 1 | 0 (0) | 0 (0) | 0 (0) | 0 (0) |
| Grade 2 | 1 (2) | 0 (0) | 1 (6) | 0 (0) |
| Grade 3 | 0 (0) | 0 (0) | 0 (0) | 0 (0) |
| Grade 4 | 0 (0) | 0 (0) | 0 (0) | 0 (0) |
